# Phonological working memory and linguistic processing speed in inferential reading comprehension

**DOI:** 10.1186/s41155-025-00356-z

**Published:** 2025-07-01

**Authors:** Daniela Balonyi Candal, Clara Regina Brandão de Avila

**Affiliations:** Department of Speech Language and Hearing Sciences, Escola Paulista de Medicina, Universidade Federal de São Paulo, São Paulo, Brazil

**Keywords:** Reading comprehension, Mental processes, Working memory, Elementary school, Processing speed

## Abstract

**Background:**

Phonological working memory has been known as an essential predictor of reading comprehension in children. However, less attention has been paid to processing speed and its interaction with working memory.

**Main body:**

Research has indicated that higher processing speed of linguistic information contributes to greater availability of memory resources used to comprehend a read text.

**Objective:**

We tested, using simple mediation models, whether phonological working memory can predict inferential reading comprehension when mediated by linguistic processing speed.

**Methods:**

To do this, we analyzed information from a database on the assessment of phonological memory (digit span Backward and Forward task), language processing speed (Verbal Fluency and Rapid Automated Naming) and inferential reading comprehension of 66 typical 5th grade students.

**Results:**

Both phonological working memory and cognitive-linguistic information processing speed were able to predict the inferential reading comprehension of students in the 5th year of elementary school. The mediation analysis showed that rapid automatized naming and working memory (digit span Backward and Forward Task) together, but independently, were able to predict inferential reading comprehension.

**Conclusion:**

When measured by semantic verbal fluency, linguistic processing speed mediated the prediction of phonological working memory (digits in Forward and Backward order) in inferential reading comprehension.

**Supplementary Information:**

The online version contains supplementary material available at 10.1186/s41155-025-00356-z.

## Highlights

1. Language processing speed (measured by Semantic Verbal Fluency) mediated the effect of phonological working memory (Forward and Backward) in inferential reading comprehension.

2. Processing speed, assessed by semantic and phonological verbal fluency, correlated with the inferential comprehension of implicit information in the text.

3. Phonological working memory contributed to processing speed, assessed by the Verbal Fluency Task.

4. The direct and independent contribution of processing speed to reading comprehension shows that this can also be an independent ability.

**Supplementary Information:**

The online version contains supplementary material available at 10.1186/s41155-025-00356-z.

## Introduction

Reading comprehension is a multifaceted process that involves various cognitive and linguistic functions. The Simple View of Reading suggests that this complexity can be understood as the result of two key skills: decoding and linguistic comprehension (Gough & Tunmer, 1986; Nation, 2019). Reading without accessing word meanings and, as a result, the ideas contained in the text cannot be considered efficient (Oakhill et al., 2019) since understanding should be its outcome. In the same direction, slow and inaccurate reading can hinder a clear understanding of what is written (Kendeou et al., 2014; Kendeou et al., 2014; Gustafson et al., 2013; Nation, 2019). Recognition becomes automatic once a word is decoded and meaning units are linked. Working memory is essential in integrating information at the phrase level (Kendeou et al., 2014).

DSM V (Diagnostic and Statistical Manual of Mental Disorders) describes reading slowness and inaccuracy as factors that may impair understanding of the text read. Therefore, reading speed, which expresses how automatic the recognition of written words is, is also related to understanding (APA, 2014). The meanings of language units proposed in a text can be combined by high-order cognitive integration and inference mechanisms (Cain et al., 2004; Kendeou et al., 2014). This process is favored by working memory, which mediates the integration of the propositions read in the text and sustains them until long-term memory information is also integrated with information from the text read (Cain et al., 2004; Kendeou et al, 2014; Novaes et al., 2019). Working memory is an essential predictor of reading comprehension in children (Cain et al., 2004; Kendeou et al., 2014; Nouwens et al., 2017; Novaes et al., 2019).

Other processing skills, like the speed of meaning activation (Oakhill et al, 2019), phonological information access (Nation, 2019), or other general ones (Gustafson et al., 2013), also contribute to reading comprehension, and the speed with which knowledge is activated may possibly be a factor that influences reading comprehension outcome (Oakhill et al., 2019). Faster information processing can mean a faster working memory rehearsal, which allows for retaining more information. Thus, linguistic information processing speed and working memory may be associated (Leonard et al., 2007) in reading comprehension.

Although the relations between processing and reading speed have also been studied, this speed is usually measured by a rapid automatized naming test and has often been associated with reading decoding, speed, and accuracy (Oliveira et al., 2014; Spätgens & Schoonen, 2018; Gerst et al., 2021). However, besides the automatic recognition of words by phonological access to the mental lexicon, the reader must quickly connect words and propositions to meanings and link previous knowledge to facilitate comprehension (Gerst et al., 2021). If the concepts of words must be retrieved from the mental lexicon (Stille et al., 2020) to provide meaning to a statement, we can question whether the accuracy and speed with which this recovery occurs favors comprehension.

Some authors consider that language components, like the mental lexicon, do the word choice to avoid repetition; vocabulary size and word knowledge are also found in the cognitive structure of the verbal fluency task (Shao et al., 2014; Whiteside et al., 2016). Thus, the analysis of the present study is necessary to verify how much these measures can contribute to the understanding of individual variation in reading comprehension (Gerst et al., 2021).

Processing speed is a broad concept, and measuring such speed still requires study. On the other hand, some ways to measure processing speed are recognized, and most of them are linked to visual stimuli (Norton & Wolf, 2012; de Oliveira et al., 2014; Gerst et al., 2021). The lack of a direct linguistic processing speed measurement procedure led us to admit the performance result in a rapid automatized naming task and (semantic and phonological) verbal fluency task as a proxy. Although we recognize their different natures, these measures were taken as the linguistic processing speed.

From these considerations, the present study investigated the contribution of working memory and the linguistic processing speed to reading comprehension regarding inferential information (Baker & Stein, 1981; Cain & Oakhill, 1999; Lúcio et al., 2015). There is consensus that the higher the speed of information processing underlying reading skills, the greater the availability of resources in memory for comprehension (Leonard et al., 2007; Language and Reading Research Consortium & Logan, 2017). The literature indicates that slow or inefficient lexical processing restricts the resources required for constructing the mental model available in working memory (Language and Reading Research Consortium & Logan, 2017). Therefore, at this moment, we decided to investigate whether these processing speed measures the relationship between working memory and reading comprehension.

When a functional level of decoding is reached, the language comprehension process, such as access to word meanings, morphosyntactic processing, integration of prior knowledge, and pragmatic processing, becomes dominant in reading comprehension (Kendeou et al., 2014; Gustafson et al., 2013; Nation, 2019). Therefore, we evaluated Brazilian fifth graders. In this stage of education, lower-level processes, such as decoding, are expected to be automatized and reading fluency achieved (BRASIL, 2018; Cogo-Moreira et al., 2023) to facilitate memory and the ability to perform inferences. The studied database was created during previous research. This research was based on the hypothesis that linguistic processing speed mediates the relationship between working memory and inferential reading comprehension in the studied sample.

## Objective

To investigate whether processing speed mediates the relationship between working memory and inferential reading comprehension in Brazilian fifth graders.

## Methods

### Sample and procedures

In a quest to understand the complex relationship between memory tasks and reading comprehension, a comprehensive study was initiated, drawing upon an anonymous database. This database was created during a previous research project, identified as 0742/2018 and accepted by Research Ethics Committee (CEP) of Universidade Federal de São Paulo, which examined “Performance in Different Memory Tasks and Their Relationships with Reading Comprehension". This present study was also accepted by CEP of Universidade Federal de São Paulo and identified as 0926/2021”

All guardians of the participants provided consent, and the children themselves expressed their assent to take part in this academic endeavor. The boards of the four schools involved also signed off on the consent forms after a thorough presentation of the original project. The research was conducted within the supportive environment of the xxxxxxxxxxx Speech Therapy Department.

The study focused on a sample of 78 monolingual (Brazilian Portuguese) fifth graders, comprising 27 boys and 51 girls, with a mean age of 10.8 years. These students were enrolled in a mix of three private schools and one public school, the latter of which had an IDEB score of 5.1 as of 2017, located in the municipality of São Paulo. Among the participants, 36 students (46%) came from private schools, while 42 (54%) were from the public school. Although the students are indeed monolingual, they are exposed to English classes in private schools.

Data regarding the participants’ gender, age, and performance in memory tasks, processing speed, reading, and reading comprehension were meticulously gathered from the assessment protocols. The evaluations were conducted during the second semester of 2018. Teachers recommended the participants, affirming that none exhibited complaints or difficulties related to reading or writing, nor were there indications of school retention, visual or auditory sensory deficits, or any cognitive, behavioral, or neurological disorders.

To ensure the quality of the sample, strict criteria were applied. Students were excluded if they did not achieve a minimum reading speed of 91 words per minute (w.p.m.) on a narrative text appropriate for fifth graders. Additionally, those who could not read at least 35 words correctly per minute in an oral test of isolated words were also removed from the sample. Accuracy was defined by a standard of fluency on the first attempt, meaning that participants read without hesitation, segmentation, or self-corrections, demonstrating automatic recognition of words.

As a result of these rigorous procedures, information about 12 children was excluded, leaving a final sample of 66 fifth graders. This final group consisted of 25 boys and 41 girls, maintaining the mean age of 10.8 years. Among them, 31 (46.97%) were from private schools, while 35 (53.03%) were from the public school system, reflecting a balanced representation of educational backgrounds within the study.

### Instruments

#### Processing speed (proxy)

We considered processing speed as the performance on the verbal fluency tasks and on the rapid automatized naming of familiar objects test.

Semantic Verbal Fluency Task—To measure the speed of lexical access by semantic cue, the participant was instructed to name as many animals and fruits in one minute (Anderson et al., 2001). The number of words produced for the semantic category, in total, was used in this study.

Phonological Verbal Fluency Task—To measure the speed of lexical access by phonological cue, the participant was instructed to name as many words as possible that started with a certain phoneme (/f/,/a/,/s/) in one minute (Anderson et al., 2001). The number of words produced for the phonological category, in total, was used in this study.

Rapid Automatized Naming Task—The speed of access to the lexicon was evaluated by the rapid naming of objects (Lúcio et al., 2017). Two boards were presented after training: board A and, soon after, board B. The errors were classified as: omissions, repetitions, and hesitations (latency above two seconds). The maximum score possible on the task was 72 points—36 possible points on each board (Lúcio et al., 2017). The number of correct answers was recorded and used in this study.

Rapid Automatized Naming (time) Task—Two boards were presented after training: board A and, soon after, board B. The total time spent in seconds was recorded and used in this study.

### Memory

Memory for Digit Span: Digits Span Forward Task (DF) – WISC-III-NL (Wechsler et al., 2002): the short-term memory span was evaluated by repeating increasing sequences of digits (from two digits) in the same order presented orally by the evaluator. The test was interrupted after an error in two attempts at the same item. The number of points corresponding to the correct answers was computed. The maximum score possible on the task was 16 points (per Figueiredo & Nascimento, 2007). The total score was used in this study.

Memory for Digit Span: Digits Span Backward Task (DB) – WISC-III-NL (Wechsler et al., 2002): the phonological working memory was evaluated by repeating increasing sequences of digits in the backward order to that presented by the evaluator. The same interruption criteria and the maximum score of the digits forward task (per Figueiredo & Nascimento, 2007) were used. The total score was used in this study.

Pseudowords Forward Repetition Task: the working phonological memory was evaluated through the Brazilian Children’s Test of Pseudoword Repetition– BCPR (Santos & Bueno, 2003). Participants were instructed to repeat the pseudowords presented orally by the evaluator. One point was awarded for each correct answer, and at the end, the total number of correct answers was computed (maximum of 40 points) and used for this study.

Verbal Episodic-Semantic Memory Task – Child Brief Neuropsychological Assessment Battery (NEUPSILIN-INF) (Salles et al., 2016) divided into immediate and late memory stages. The evaluator presented a list of nine words orally, and participants were instructed to repeat all the words they remembered following the evaluator. After some time, the evaluator asked the participant again to repeat the words that were heard. The total number of correct words repeated in immediate and delayed recall was computed in this study.

### Reading comprehension

Reading comprehension of expository text (The Anteater) Task (Lúcio et al., 2015)—Participants received the following instruction: “You must read this text how you are used to it. Try to understand the text as best you can. If you understand well by reading aloud, do so. If you understand better by reading silently, then do so. The most important thing is that you read it carefully because you will answer some questions after reading it. May we start?” After reading, comprehension was verified through 15 open-ended questions proposed orally to the participants. Questions were sorted into three categories, each assessing different cognitive processes of comprehension: literal; inferential of global coherence; and local cohesion (Cain & Oakhill, 1999; Lúcio et al., 2015). The responses were recorded and later transcribed by the evaluator. Correct and wrong answers were computed based on the responses. One point was awarded for each correct answer (a maximum of 15 points). Once inferential comprehension was the object of study, only the number of points for understanding inferential questions was used in this study.

### Statistical methodology

The mediation analysis began with investigating the direct effect of working memory on the prediction of processing speed *(a)*. Subsequently, the direct effects of processing speed *(b)* and working memory *(c’)* on reading comprehension were analyzed independently. Finally, we analyzed the indirect effect of working memory by processing speed *(ab)* on reading comprehension. The adjustment measures (*R*^2^ value) were compared between the models that calculated the independent effect *(b and c’)* and the total effect *(ab* + *c’)* of variables in predicting reading comprehension for those models in which there was evidence of an indirect effect (i.e., mediation).

Data were analyzed using descriptive and inferential statistics. Minimum and maximum values, mean, and standard deviation were described for each performance measure of linguistic working memory, processing speed, and reading comprehension tasks. The correlation between the study variables was analyzed using Pearson’s r coefficient, with values of 0.10, 0.30, and 0.50 considered weak, moderate, and strong effects, respectively (Cohen, 1988).

Data were analyzed using the Statistical Package for Social Science ([SPSS], version 22.0). The PROCESS macro version 4.0 (https://www.processmacro.org/) was used for the mediation analysis. Values of *p* ≤ 0.05 and confidence intervals that did not contain zero between the upper and lower limits were considered significant. Confidence intervals (95% CI) were generated using bootstrap percentiles with 5000 replications.

**Fig. 1:**
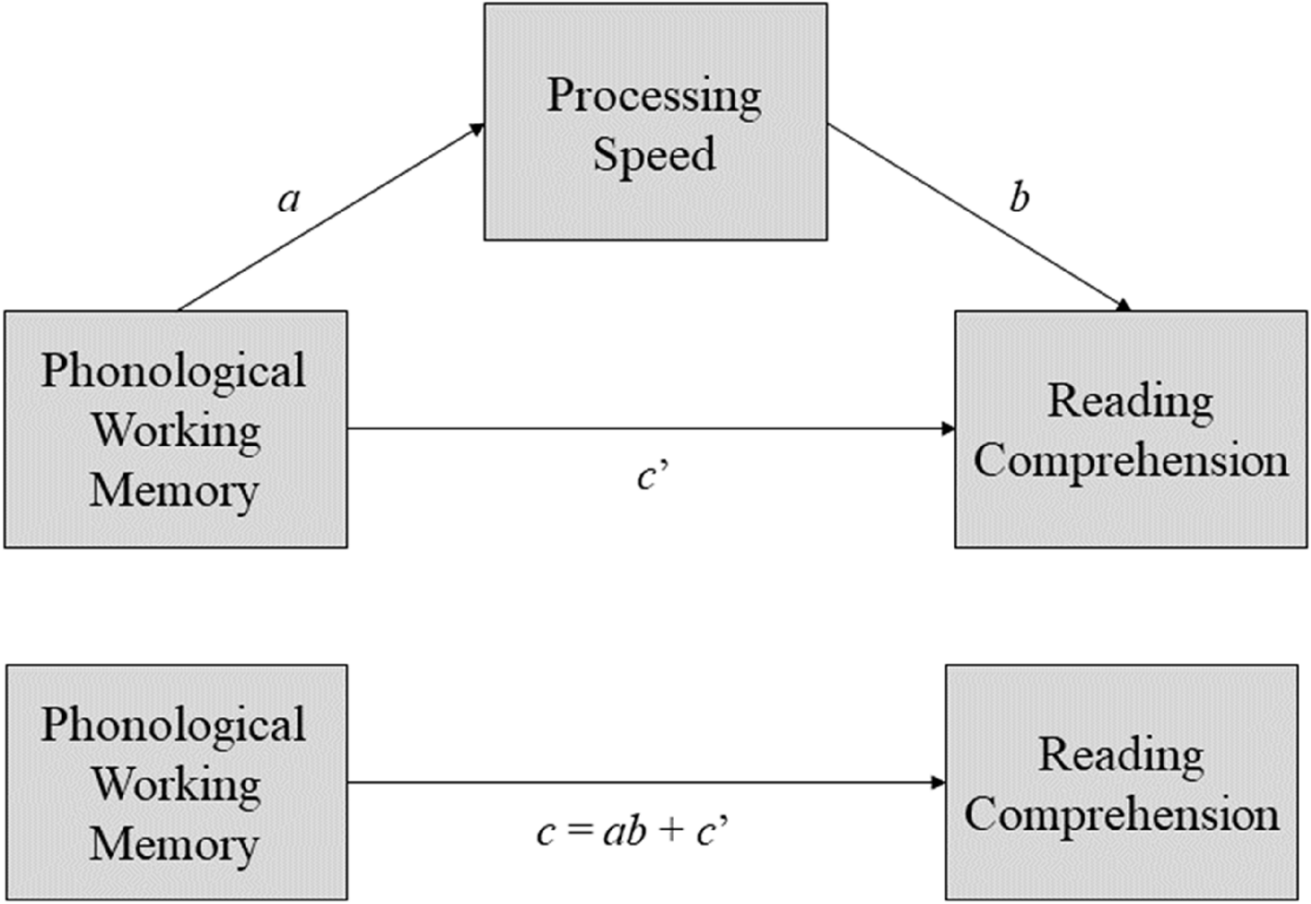
Theoretical model of simple mediation of working memory by processing speed in reading comprehension. Note: *a*, direct effect of working memory on processing speed; *b*, direct effect of processing speed on reading comprehension; *c'*, direct effect of working memory on reading comprehension; *ab*, indirect effect (mediated); *c*, total effect. Adapted from Hayes (2022)

Simple mediation models were tested to verify the hypothesis that predicting reading comprehension by linguistic working memory is mediated by processing speed (Model 4, Hayes, 2020). We included working memory and processing speed measures that obtained a value of *r* ≥ 0.20 (i.e., correlations close to the moderate effect) or significant effects (*p* ≤ 0.05) in the correlation analysis with reading comprehension. The working memory performance variables were tested as antecedent variables, and the processing speed variables as mediators. Figure 1 describes the theoretical model of the tested models.

Bonferroni correction was used to control *p*-value inflation, in which the significance level (0.05) was divided by the number of models compared.

### Data availability

https://osf.io/nfkxp/?view_only=9dc577987d1c414190d168b94c872ab6.

## Results

Descriptive performance statistics on processing speed, working memory, and reading comprehension tasks are summarized in Table 1.

**Table 1:** Descriptive statistics of processing speed, working memory, and reading comprehension measures

Variable	Minimum	Maximum	Mean	SD
**Working memory**				
Digits Span Forward Task	4	11	7.14	1.50
Digits Span Backward Task	2	7	3.98	1.26
Forward Repetition of Pseudowords Task	33	40	38.33	1.67
Immediate Memory	2	9	5.18	1.49
Late Memory	0	8	3.89	1.60
**Processing speed**				
Rapid Automatized Naming (Hits)	57	72	66.47	3.30
Rapid Automatized Naming (Time)	43	77	57.86	8.47
Semantic Fluency	16	41	27.24	5.20
Phonological Fluency	2	32	20.27	7.04
**Reading comprehension**				
Inferential Comprehension	1	11	4.65	2.16

*SD* standard deviation. Rapid naming (Time) = Seconds. Semantic verbal fluency = Total words evoked in one minute by semantic cues in the Verbal Fluency task. Phonological verbal fluency = Total words evoked in one minute by phonological cues in the Verbal Fluency task

The correlations between working memory, processing speed, and reading comprehension measures are shown in Table 2. We found significant correlations from weak to moderate effect (*r* = 0.25 to *r* = 0.38) of inferential and total reading comprehension with working memory and processing speed measures. No significant correlation was found between the variables of correct answers in the rapid naming task and literal reading comprehension.

**Table 2:** Correlation between processing speed, working memory, and reading comprehension measures

Variables	Processing speed				Working memory					Inferential Reading Comprehension
	1	2	3	4	5	6	7	8	9	10
1. Rapid Automatized Naming (Hits)	-									
2. Rapid Automatized Naming (Time)	-.58**	-								
3. Semantic Fluency	.16	-.17	-							
4. Phonological Fluency	.08	-.06	.34*	-						
5. DF	.17	-.13	.26*	.07	-					
6. DB	.12	-.17	.30*	.16	.30*	-				
7. Forward Repetition of Pseudowords	-.19	.27*	-.18	.02	.05	-.11	-			
8. Immediate Memory	-.12	-.13	.16	.05	.30*	.27*	-.13	-		
9. Late Memory	.11	-.06	.14	-.01	.14	.04	-.50	.67**	-	
10. Inferential Reading Comprehension	.16	-.28*	.35**	.25*	.32**	.38**	.06	.08	-.02	-

**Significant correlation (< 0.0001). *Significant correlation (0.05). Time = Seconds. DF Digit Span Forward Task, DB Digit Span Backward Test. Rapid naming (Time) = Seconds. The Verbal Episodic-Semantic Memory task evaluated Immediate Memory and Late Memory. Semantic fluency = Total words evoked in one minute by semantic cues in the Verbal Fluency task. Phonological fluency = Total words evoked in one minute by phonological cues in the Verbal Fluency task

Greater inferential reading comprehension scores were associated with a shorter rapid naming time (*r* = − 0.28 and *r* = − 0.29, respectively) and higher scores in the semantic fluency tasks (*r* = 0.35 and *r* = 0.32, respectively). Furthermore, greater inferential comprehension was related to higher scores in the semantic fluency task (*r* = 0.25).

Regarding working memory measures, greater inferential comprehension and total ability were associated with higher scores in the digits span forward task (DF) (*r* = 0.32 and r = 0.34, respectively) and digits span backward task (DB) (*r* = 0.38 and *r* = 0.36, respectively). Finally, a longer rapid naming time was related to a more significant number of repeated pseudowords (*r* = 0.27). A more significant number of words produced by semantic cues was related to higher scores in DF (*r* = 0.26) and DB (*r* = 0.30) digits.

### Inferential reading comprehension

Six simple mediation models were tested with working memory measures (forward and backward order digits) as antecedent variables and processing speed measures that obtained an *r* value ≥ 0.20 as mediators (i.e., correlations close to the moderate effect) or significant effects (*p* ≤ 0.05) in the correlation analysis with reading comprehension (rapid naming time and word evocation by semantic and phonological cues). Rapid Automatized Name (Hits), Forward Repetition of Pseudowords, and Immediate and Late Memory were not included in mediation models because they did not obtain significant effects in the correlation analysis with reading comprehension.

### Model 1: Rapid naming time as a mediator and working memory evaluated by the performance on the digits span forward task

**Fig. 2:**
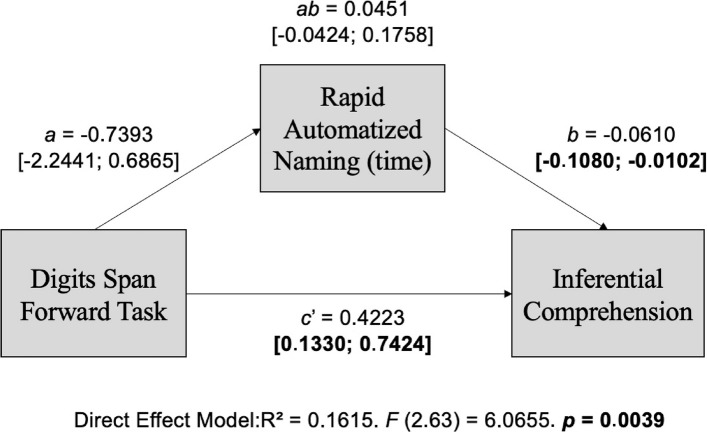
Theoretical model of the direct and indirect effect of working memory (Digits Span Forward Task) and processing speed (Rapid Automatized Naming Time) on inferential reading comprehension. Note: *a*, direct effect of working memory on processing speed; *b*, direct effect of processing speed on reading comprehension; *c’*, direct effect of working memory on reading comprehension; *ab*, indirect effect (mediated). Adapted from Hayes (2022)

The first mediation model tested aimed at evaluating the indirect effect of a rapid naming task on the influence of a digit span forward task during inferential reading comprehension. For this purpose, rapid naming time was used as a processing speed measure, and the performance on the digit span forward task (DF) as a working memory measure (Fig. 2).

There was no indirect effect of working memory on processing speed (*ab* = 0.0451; [− 0.0424; 0.1758]), which indicates the lack of a mediating effect of processing speed on the relationship between working memory (measured by DF) and comprehension. However, the direct effect of working memory on comprehension remained significant (*c’* = 0.4223, [0.1330; 0.7424]). The impact of processing speed on comprehension was also significant in isolation [*b* = − 0.0610; [− 0.1080; − 0.0102]) (Table 3).

**Table 3:** Model 1 Coefficients: Phonological Working Memory (Digits Span Forward Task) and Processing Speed (Rapid Automatized Naming—time) in the inferential reading comprehension

Variables	Processing speed (RN Time)				Inferential comprehension			
		Coefficient	SE	*p*		Coefficient	SE	*p*
Working memory (DF)	*a*	− 0.7393	0.7010	0.2955	*c'*	0.4223	0.1678	0.0144
Processing speed (RAN Time)					*b*	−0.0610	0.0297	0.0438
Constant		63.1397	5.1097	0.0001		5.1692	2.2309	0.0238
		*R*^2^ = 0.0171 *F* (1.64) = 1.1124. *p* = 0.2955				*R*^2^ = 0.1615 *F* (2.63) = 6.0655. *p* = 0.0039		

a, direct effect of working memory on processing speed; b, direct effect of processing speed on reading comprehension; c’, direct effect of working memory on reading comprehension

### Model 2: Rapid Automatized Naming Time as a mediator and working memory evaluated by the performance on the digits span backward task (DB)

**Fig. 3:**
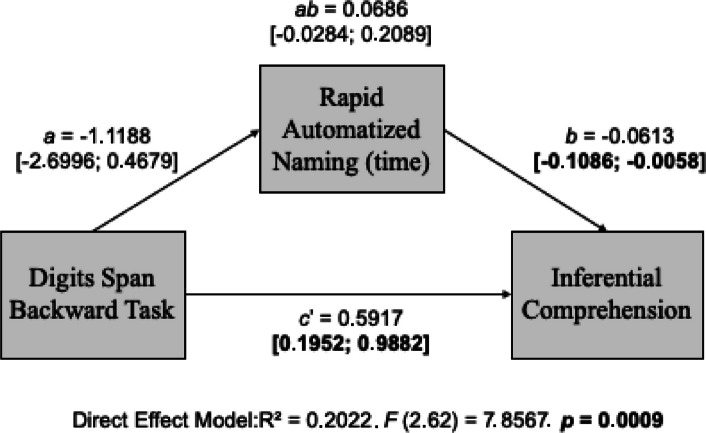
Theoretical model of the direct and indirect effect of Phonological Working Memory (Digits Span Backward Task) and processing speed (Rapid Automatized Naming Time) on Inferential Reading Comprehension. Note: *a*, direct effect of working memory on processing speed; *b*, direct effect of processing speed on reading comprehension; *c’*, direct effect of working memory on reading comprehension; *ab*, indirect effect (mediated). Adapted from Hayes (2022)

Model 2 aimed to evaluate the indirect effect of processing speed on the influence of a digit span backward task on inferential comprehension. This model evaluated processing speed by rapid naming time and working memory by the digit span backward task (DB)(Fig. 3).

There was no indirect effect of processing speed on inferential reading comprehension (*ab* = 0.0686, [− 0.0284; 0.2089]), indicating no mediator effect. Working memory (*c’* = 0.5617, [0.1952; 0.9882]) and processing speed (*b* = − 0.0613, [− 0.1086; − 0.0058]) had a significant independent impact on inferential comprehension (Table 4).

**Table 4:** Model 2 coefficients: Working Memory (Digits Span Backward Task) and Processing Speed (Rapid Automatized Naming—time) in the inferential reading comprehension

Variables	Processing speed (RN Time)				Inferential comprehension			
		Coefficient	SE	*p*		Coefficient	SE	*p*
Working memory (DB)	*a*	− 1.1188	0.8353	0.1852	*c'*	0.5917	0.1984	0.0041
Processing speed (RAN Time)					*b*	−0.0613	0.0295	0.0419
Constant		62.4735	3.4872	0.0001		5.8755	2.0159	0.0049
		*R*^2^ = 0.0277 *F* (1.64) = 1.7942. *p* = 0.1852				*R*^2^ = 0.2022 *F* (2.63) = 7.8567. *p* = 0.0009		

Note: a, direct effect of working memory on processing speed; b, direct effect of processing speed on reading comprehension; c’, direct effect of working memory on reading comprehension

### Model 3: Word evocation by semantic cues as a mediator and working memory assessed by the performance on the digits span forward task (DF)

**Fig. 4:**
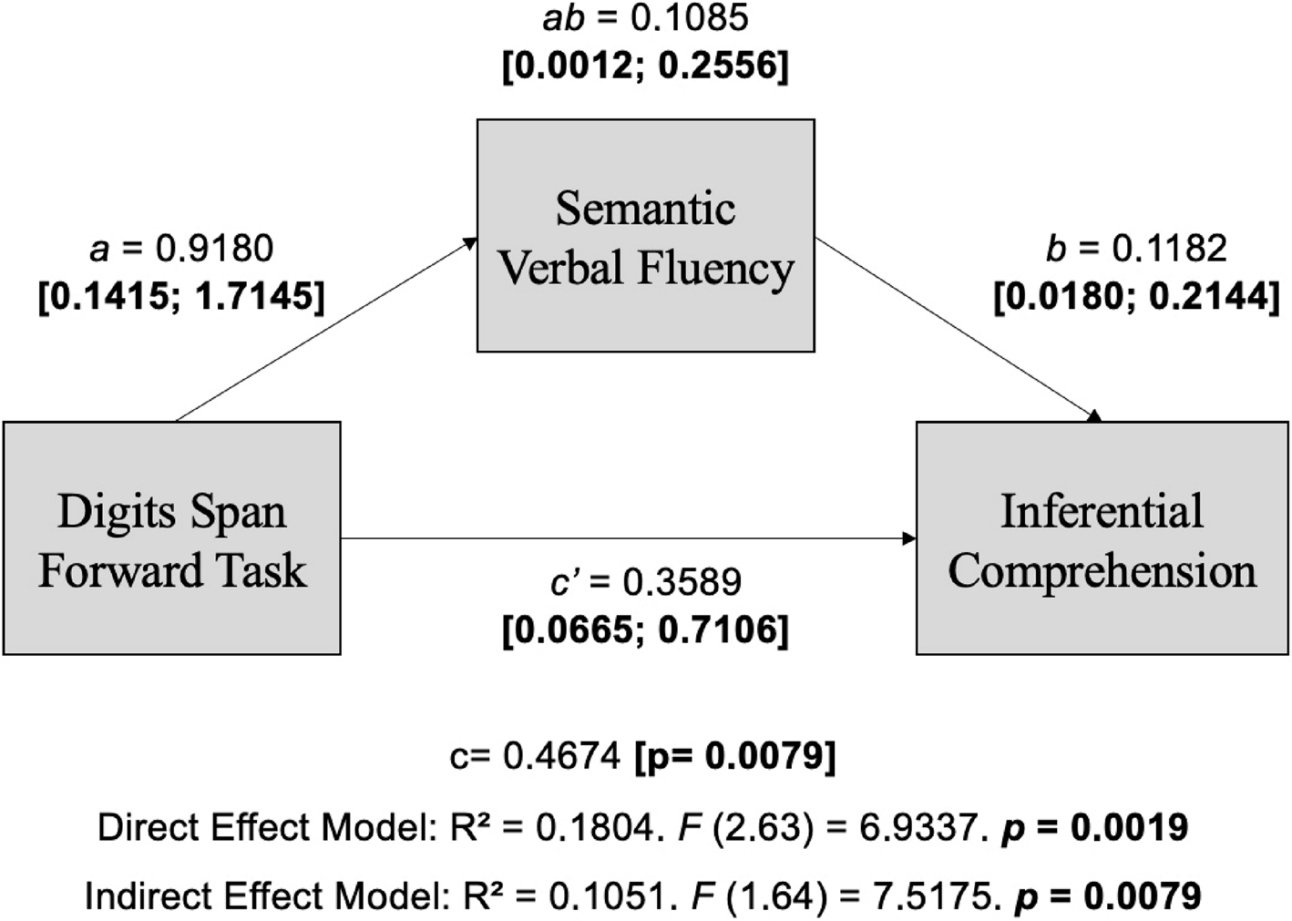
Theoretical model of the direct and indirect effect of Phonological Working (Digits Span Forward Task) and Processing Speed (Semantic Verbal Fluency) on inferential reading comprehension. Note: *a*, direct effect of working memory on processing speed; *b*, direct effect of processing speed on reading comprehension; *c’*, direct effect of working memory on reading comprehension; *ab*, indirect effect (mediated); c, total effect. Adapted from Hayes (2022)

Model 3, which evaluated the indirect effect of processing speed on the influence of a digit span forward task on inferential reading comprehension, had word evocation by semantic cues (semantic fluency) as a measure of processing speed and working memory measured by the digit span forward task (Fig. 4).

We observed an indirect effect of processing speed on the influence of a digit span forward task on inferential comprehension (*ab* = 0.1085, [0.0012; 0.2556]), suggesting a mediating effect of processing speed (semantic verbal fluency) on inferential reading comprehension. An increase in working memory span was directly associated with increased processing speed (*a* = 0.9180, [0.1415; 1.7145]). In isolation, processing speed (*b* = 0.1182, [0.0180; 0.2144]) and working memory (*c’* = 0.3589, [0.0665; 0.7106]) directly impacted inferential reading comprehension. Model 3 parameters are shown in Table 5.

**Table 5:** Coefficients Model 3: Phonological Working Memory (Digits Span Forward Taks) and Processing Speed (Semantic fluency) in the inferential reading comprehension

Variables	Processing Speed (Semantic fluency)				Inferential Comprehension			
		Coefficient	SE	*p*		Coefficient	SE	*p*
Working memory (DF)	*a*	0.9180	0.4184	0.0319	*c'*	0.3586	0.1705	0.0392
Processing speed (Semantic fluency)					*b*	0.1182	0.0491	0.0191
Constant		20.6913	3.0500	0.0001		−1.1295	1.5716	0.4750
		*R*^2^ = 0.0700 *F* (1.64) = 4.8136. *p* = 0.0319				*R*^2^ = 0.1804 *F* (2.63) = 6.9337. *p* = 0.0019		

a, direct effect of working memory on processing speed; b, direct effect of processing speed on reading comprehension; c’, direct effect of working memory on reading comprehension

### Model 4: Word evocation by semantic cues as a mediator and working memory evaluated by digits span backward task (DB)

**Fig. 5:**
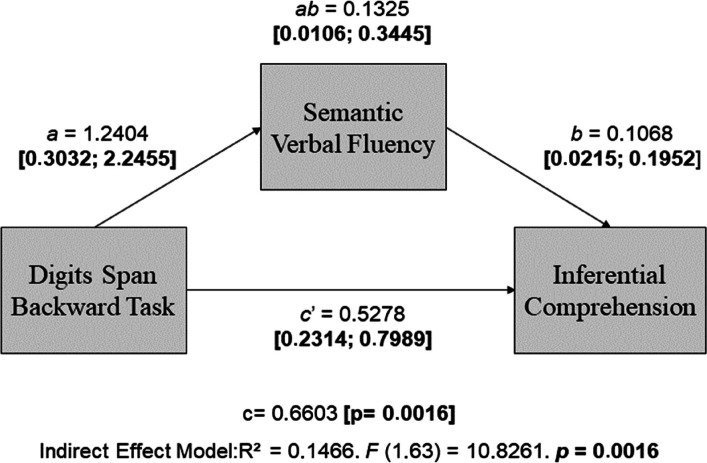
Theoretical model of the direct and indirect effect of Phonological Working Memory (Digits Span Backward Task) and Processing Speed (Semantic Verbal Fluency) on inferential reading comprehension. Note: *a*, direct effect of working memory on processing speed; *b*, direct effect of processing speed on reading comprehension; *c’*, direct effect of phonological working memory on reading comprehension; *ab*, indirect effect (mediated); c, total effect. Adapted from Hayes (2022)

Model 4 was tested to analyze the indirect effect of processing speed (assessed by semantic fluency) on the influence of a digit span backward task on inferential reading comprehension (Fig. 5).

We identified an indirect effect of processing speed on working memory in inferential comprehension (*ab* = 0.1325, [0.0106; 0.3445]), indicating a mediating effect of processing speed (semantic fluency) on working memory (digits span backward task) in inferential comprehension. Increasing working memory was associated with increased processing speed (*a* = 1.2404, [0.3032; 2.2455]). Processing speed (*b* = 0.1068, [0.0215; 0.1952]) and working memory (*c’* = 0.5278, [0.2314; 0.7989]) alone impacted inferential reading comprehension performance as well. Model 4 parameters are shown in Table 6.

**Table 6:** Coefficients Model 4: Phonological Working Memory (Digits Span Backward Task) and Processing Speed (Semantic fluency) in the inferential reading comprehension

Variables	Processing speed (Semantic fluency)				Inferential comprehension			
		Coefficient	SE	*p*		Coefficient	SE	*p*
Working memory(DB)	*a*	1.2404	0.5014	0.0161	*c'*	0.5278	0.2045	0.0121
Processing speed (Semantic fluency)					*b*	0.1068	0.0490	0.0331
Constant		22.3190	2.0932	0.0001		−0.3376	1.3631	0.8052
		*R*^2^ = 0.0886 *F* (1.64) = 6.1210. *p* = 0.0161				*R*^2^ = 0.2074 *F* (2.63) = 8.1117. *p* = 0.0007		

a, direct effect of working memory on processing speed; b, direct effect of processing speed on reading comprehension; c’, direct effect of working memory on reading comprehension

### Model 5: Word evocation by phonological cues as a mediator and working memory assessed by digits span forward task (DF)

Model 5 evaluated the indirect effect of processing speed (phonological fluency) on the influence of phonological working memory (assessed by digits span forward task) on inferential comprehension. There was no indirect effect of processing speed in predicting reading comprehension (*ab* = 0.0225, [− 0.0817; 0.1095]). Working memory (DF), in isolation, influenced reading comprehension (*c’* = 0.4449, [0.1689; 0.7740]).

**Table 7:** Coefficients Model 5: Phonological Working Memory (Digits Span Forward Task) and Processing Speed (Phonological fluency) in the inferential reading comprehension

Variables	Processing speed (Phonological fluency)				Inferential comprehension			
		Coefficient	SE	*p*		Coefficient	SE	*p*
Working memory (DF)	*a*	0.3193	0.5859	0.5876	*c'*	0.4449	0.1671	0.0098
Processing speed (Phonological fluency)					*b*	0.0704	0.0356	0.0521
Constant		17.9941	4.2706	0.0001		0.0486	1.3735	0.9719
		*R*^2^ = 0.0046 *F* (1.64) = 0.2970. *p* = 0.5876				*R*^2^ = 0.1575 *F* (2.63) = 5.8902. *p* = 0.0045		

a, direct effect of working memory on processing speed; b, direct effect of processing speed on reading comprehension; c’, direct effect of working memory on reading comprehension

### Model 6: Word evocation by phonological cues as a mediator and working memory evaluated by digits span backward task

The last model, Model 6, evaluated the indirect effect of processing speed, assessed by phonological fluency, on the influence of working memory (digits span backward task) on inferential reading comprehension. This model had no indirect effect (*ab* = 0.0504, [− 0.0274; 0.1851]). Isolated phonological working memory impacted the sample’s inferential reading comprehension performance (*c’* = 0.6099, [0.2880; 0.9137]).

**Table 8:** Coefficients Model 6: Phonological Working Memory (Digits Span Backward Task) and Processing Speed (Phonological Fluency) in the inferential reading comprehension

Variables	Processing speed (Phonological fluency)				Inferential comprehension			
		Coefficient	SE	*p*		Coefficient	SE	*p*
Workingmemory (DB)	*a*	0.8751	0.6980	0.2146	*c'*	0.6099	0.2006	0.0035
Processingspeed (Phonologicalfluency)					*b*	0.0576	0.0358	0.1127
Constant		16.8824	2.9143	0.0001		1.0744	1.0244	0.2983
		*R*^2^ = 0.0243 *F* (1.64) = 1.5716. *p* = 0.2146				*R*^2^ = 0.1808 *F* (2.63) = 3.9665. *p* = 0.0021		

a, direct effect of working memory on processing speed; b, direct effect of processing speed on reading comprehension; c’, direct effect of working memory on reading comprehension.

## Discussion

Reading comprehension is a complex task with different cognitive processes that lead to the formation of mental representations of the information read (Cain & Oakhill, 1999; Kendeou et al., 2020). Working memory is a multi-component system, particularly relevant for text reading comprehension (Baddeley, 2003; Oakhill et al., 2019). It preserves information while cognitive processes are carried out, integrating them or incorporating them into previous knowledge to make sense of the implicit details (Oakhill et al., 2019).

Although this importance is recognized, more recently, the literature is also beginning to discuss the contribution of processing speed to the mechanisms involved in reading fluency and comprehension (Gerst et al., 2021). Processing speed can refer to a variety of perceptual and cognitive components, and by being a quantity can be measured by different time scales (Gerst et al., 2021).

Some authors consider rapid automatized naming tests a task to measure the processing speed of linguistic information (Norton & Wolf, 2012; Oliveira et al., 2014; Gerst et al., 2021, Spätgens & Schoonen, 2018). It is often associated with reading fluency (Papadopoulos, Spanoudis & Georgiou, 2016). It is often associated with reading fluency (Papadopoulos, Spanoudis & Georgiou, 2016).

In this study, the verbal fluency test, measuring the number of evoked words per minute (words/ΔT) was considered a second measure of cognitive-linguistic processing speed (Table 1). In fact, among several processing tasks, Gerst et al. (2021) considered the possibility that verbal fluency provides speed and accuracy measures. Therefore, both the speed of immediate lexical access by visual stimulus and the speed of lexical access by evoking categorized and long-term memory were taken as proxies of the processing speed of linguistic information (Table 1). We aimed to investigate whether processing speed mediates the relationship between working memory and inferential reading comprehension in Brazilian fifth graders.

In the analyses, we found evidence of a correlation between phonological working memory, assessed by repeating digits in forward and backward order, and linguistic processing speed, assessed by semantic verbal fluency (Table 2). The literature recognizes this relationship between processing speed and phonological working memory (Leonard, 2007), although it discusses more widely and more frequently the influence of memory on reading comprehension (Cain et al., 2004; Kendeou et al., 2014; Nouwens et al., 2017; Castles et al., 2018; Novaes et al., 2019; Oakhill et al., 2019) and less on how the processing speed of linguistic information can affect connections between written words and their meanings, and consequently, reading comprehension (Gerst et al., 2021).

From the speed perspective, information will be susceptible to degradation, decline, or even interference from new information (Leonard et al., 2007; Park et al., 2015) if it is not quickly processed. Therefore, the higher processing speed should allow faster rehearsal (Leonard et al., 2007) and, consequently, more information to be consolidated more quickly. Similarly, it should allow the allocation of more cognitive resources to contribute to comprehension. According to Norton and Wolf (2012), when the perceptual, cognitive, and linguistic processes involved in the comprehension levels (sublexical, words, and connected text) are processed automatically (i.e., with speed and precision), time and resources can be directed to allow a deeper comprehension of the text read.

These theoretical concepts support the findings of this study, which aimed to understand whether the contribution of phonological working memory to inferential reading comprehension is mediated by the processing speed in the fifth grade of Elementary School. The main results from this study were as follows: (1) processing speed, measured by semantic verbal fluency, mediated the effect of phonological working memory in inferential reading comprehension (Tables 1 and 7); (2) there was no mediating effect of processing speed, measured by rapid naming speed, on the effect of phonological working memory on reading comprehension (Tables 5 and 6); (3) the direct and independent effect of phonological working memory and processing speed measured by rapid automatized naming time (Figs. 3 and 4; Tables 3 and 4) and verbal fluency (Figs. 4 and 5 ; Tables 5 and 6) explained the inferential comprehension performance variance.

This study confirms that preserving information in working memory (Cowan & Morey, 2018) is relevant for integrating them, allowing comprehension (Oakhill et al., 2019). The analysis showed that phonological working memory, assessed by repeating digits in forward and backward order, could predict the inferential comprehension of implicit information in the text and in the study by Van den Bosch et al. (2018). Likewise, processing speed, assessed by semantic and phonological verbal fluency, correlated with the inferential comprehension of implicit information in the text (Table 2).

This study found no indirect contribution of phonological working memory to the variance of inferential reading comprehension when mediated by rapid automatized naming time. However, it identified predictive reading comprehension values for these variables jointly but independently (Figs. 2 and 3; Tables 3 and 4). Gerst et al. (2021) also observed that a higher speed of rapid automatized naming contributes to reading comprehension.

Furthermore, only the measurement of naming time, not correct answers, was considered a speed measure. From a mechanism viewpoint, naming activates mental representations of appropriate phonological forms, in this case, of the words read, which connects concepts to phonological representation to achieve meaning. Activating these representations in the mental lexicon facilitates oral (Stille et al., 2020) and reading comprehension. Figure 3 and Table 4 shows that the faster a person can encode/activate phonological representations, the more text they can read and the ability to assign meaning to text will be more available (Christopher et al., 2012).

The investigation of linguistic information processing speed by semantic verbal fluency task showed a direct effect and mediated relationship between phonological working memory and inferential comprehension of the expository text read (Tables 5 and 6). In the model that considered the mediator effect, phonological working memory contributed to processing speed, assessed by the semantic verbal fluency task. The correlation analysis associated higher processing speed with a longer phonological working memory span (Table 2). These findings can raise some questions that were not answered in this study and that indicate the need for future studies to answer them: could we assume that the phonological memory span facilitates performance in the verbal fluency task? Is it possible that greater memorization capacity contributes to the quality and number of items evoked in the verbal fluency task, which involves monitoring the items already evoked? (Tables 5 and 6).

Besides confirming the hypothesis that the relationship between phonological working memory and reading comprehension is mediated by processing speed, these two variables’ direct and independent effect also seems to explain the variance in the sample’s comprehension performance (Tables 5 and 6). The direct and independent contribution of processing speed to reading comprehension shows that this can also be an independent ability. It certainly is not entirely memory-dependent; however, what other skills would contribute to the increased processing speed? Could vocabulary and its quality features be one of them?

The fact that semantic verbal fluency presented a significant mediating effect suggests that this test may be capturing another dimension or domain closer to semantic skills, such as vocabulary. This possibility better explains the inferential reading comprehension variance than phonological processing speed measures.

This possibly is pertinent since Albinet (2016) believes processing speed can be affected by other brain functions, but it can also affect them. In a study investigating whether processing speed or vocabulary size predicts later language improvement in young children, Peter et al. (2019) pointed out that higher processing speed promoted vocabulary and syntax improvement. These findings allow us to think that, just as memory contributes to greater processing speed, the opposite direction of this relationship would also be possible. It is good to remember that both occur simultaneously.

This study did not observe these relationships between working memory and processing speed when the latter was evaluated by rapid automatized naming, which shows that rapid naming and verbal fluency have different natures. However, both tasks allow for measuring cognitive-linguistic processing speed. The former evaluates automatized and immediate access to the mental lexicon, and the latter remote access.

Phonological verbal fluency and rapid automatized naming did not show mediation. It should be considered that rapid automatized naming is serial and the demand for semantic access is lower since the words have no connection and the figures shown are repeated until the end of the test. On the other hand, semantic verbal fluency, which showed mediation, accesses the mental lexicon through a semantic network.

Although the tasks of semantic and phonological verbal fluency are similar, the cognitive processes and the brain substrate underlying each seem to be different. For the semantic verbal fluency task, the generation of semantic associations and the meaning of words is fundamental to word retrieval. The phonological verbal fluency task requires greater executive control since it involves an effort in selecting words according to the initial phoneme, rather than lexical-semantic networks (Alvarez et. al, 2023). Another difference is the brain regions involved in each task, with semantic verbal fluency being related to the higher temporal lobe and phonological verbal fluency being related to the higher frontal lobe (Alvarez et. al, 2023; Kircher et. al, 2011).

In this study, phonological working memory, assessed through the Verbal Episodic-Semantic Memory Task (immediate and late memory), did not show a correlation with inferential reading comprehension. One possible explanation is that the Verbal Episodic-Semantic Memory Task (immediate and delayed recall) required participants to repeat a list of nine words, which may exceed the limits of what Unsworth and Engle (2006) refer to as primary memory. According to Unsworth and Engle (2007), primary memory is responsible for maintaining distinct representations active in consciousness for processing, with a typical span limit of about four items. When this limit is surpassed, items are displaced into secondary memory and must be retrieved via a cue-dependent search process (e.g., temporal, contextual, categorical cues) (Unsworth & Engle, 2007). It is possible that the displacement of items from primary to secondary memory is smaller in digit span tasks (forward order) than in immediate and late memory tasks.

However, when the task involves item manipulation, such as in the backward digit span task, secondary memory is also engaged (Rose et al., 2010; Unsworth & Engle, 2006). One possible explanation for the absence of a correlation between semantic memory and reading comprehension lies in the fact that retrieval from secondary memory depends on cues based on contextual elements. According to Unsworth and Engle (2007), each encoded item is associated with at least one contextual element. The variety of items presented in the immediate and late memory conditions of the Verbal Episodic-Semantic Memory Task, combined with the lack of semantic relation between them, may broaden the scope of the search set, positioning this task at a lower hierarchical level. In contrast, the backward digit span task, which involves repetition of items from the same semantic category, may place the task at a higher hierarchical level (Unsworth & Engle, 2007). In the context of this study, it is plausible to assume that the hierarchical level required to perform the tasks influenced the predictive capacity of phonological working memory in relation to reading comprehension.

Repetition of pseudowords also showed no correlation with inferential reading comprehension. This task can be distinguished from digit span tasks (both forward and backward) by the nature of the stimuli. Pseudoword repetition relies on the quality of the phonological substrate, and the stimuli are unpredictable, unlike digit repetition tasks (De Carvalho et al., 2014).

In this sense, future studies should be conducted to deepen the relationships between processing speed, memory, and reading comprehension.

Thinking about the interactions between working memory and processing speed, we could look at the possible cognitive processes involved in preserving information in working memory: consolidation, update, and deletion (Morey & Cowan, 2018). Consequently, the more information is consolidated, the less updating is required for a representation to be preserved (Morey & Cowan, 2018). Also, the faster information is processed, the less susceptible it will be to degradation or interference from new information in memory (Leonard et al., 2007; Park et al., 2015).

Therefore, despite being different skills, we could ask whether the speed and accuracy of linguistic processing can be involved in preserving information in working memory until the resolution of the reading comprehension processes, or whether they would facilitate these processes (Morey & Cowan, 2018). Gerst et al. (2021) affirm that the more a processing speed measure captures more cognitively complex processes, as in the case of verbal fluency, the more relevant it becomes to consider that there is overlap with other cognitive processes, such as executive functions. Since working memory is an executive function, it contributed to the speed of linguistic processing in the semantic verbal fluency task in this study (Tables 5 and 6).

That said, and considering that working memory tasks can reveal limited processing capacity (Leonard et al., 2007), we could question whether working memory task analyses should also consider the time factor, the processes, and the skills involved and their implications on the answers found in the memory tasks, and whether the analyses on the semantic verbal fluency task should consider the performance on the working memory task.

It is evident that the sample size interfered with, limited many statistical analyses, and hampered the analysis of a greater number of variables. Despite this, this study raises important questions and implications for future research.

One of the implications concerning clinical practice is that, as it is a simple measure widely used in speech-language and neuropsychological assessments, the verbal fluency test should be considered in reading assessments of fifth-graders. Unlike the rapid naming task, complex processing speed tasks such as verbal fluency align better with the terms “information processing” or “cognitive processing speed” (Gerst et al., 2021).

This condition also instigates future studies that deepen knowledge about the processing speed of linguistic information and its associations with other cognitive skills, including reading comprehension. We could also suggest that future studies be conducted to verify the effects of stimulating the processing speed of linguistic information on reading comprehension.

Although the results have shown that some variables have a mediating effect on predicting reading comprehension, we should emphasize that the results of this study should only be generalized within this sample since they do not allow causal inferences.

## Limitations and implications

One of the limitations of the study was not verifying during data collection information about the participants'familiarity with the subject presented in the reading text. Therefore, since this information was absent from the database, it was not considered a study variable.

The participants were enrolled in schools from a single city, which limited the sample size and prevented the generalization of the data.

A third limitation is that the study only considered children from the 5th grade of elementary school.

The possibility of a new capacity, such as processing speed, interfering with reading comprehension should be considered in future research and in the clinical analysis of reading comprehension assessment.

Another issue to be considered in future studies would be to investigate possible differences between the performances of students in public and private schools, thinking about information that could contribute to the adoption of public policies for Education.

## Conclusion

In this experiment, only cognitive-linguistic processing speed, estimated by the speed of lexical recall by semantic cue, was able to mediate the prediction of working memory in inferential reading comprehension. These results show that not only memory but also processing speed should be considered in the assessment of reading comprehension.

When considering the complexity of the mechanisms and processes involved in reading comprehension, this study focused only on these variables. In future studies, other skills, such as vocabulary and grammar, that are also established predictors of reading comprehension, should be explored.

## Supplementary Information

Supplementary Material 1.

## Data Availability

https://osf.io/nfkxp/?view_only=9dc577987d1c414190d168b94c872ab6
