# Supplementary material for: Phonological working memory and linguistic processing speed in inferential reading comprehension
Source: Psicol Reflex Crit. 2025 Jul 1;38:20. doi: 10.1186/s41155-025-00356-z (PMC12214213; doi:10.1186/s41155-025-00356-z)
Supplement: Supplementary file 1 — Supplementary Material 1. [file 41155_2025_356_MOESM1_ESM.docx]

**rEADING cOMPREHENSION**

**Texto 4 (A)**

O tamanduá-bandeira é um mamífero normalmente encontrado em florestas úmidas, campos e cerrados. Em geral, é visto acompanhado somente na primavera. A gestação do tamanduá-bandeira dura 190 dias (pouco mais de seis meses), e, depois de nascido o filhote, a fêmea cuida dele sozinha, amamentando-o por nove meses.

O corpo do tamanduá-bandeira é coberto de pelo bem grosso e seu rabo parece um espanador de pó. Seu focinho é fino e longo e sua boca não tem dentes. Ele gosta de formigas e cupins e procura comida o tempo todo. Usa as garras grandes e afiadas, das patas dianteiras, para cavar formigueiros e quebrar cupinzeiros. Outra ferramenta importante é sua língua comprida e grudenta, pois, após cavar o mais fundo que pode, é com ela que alcança o alimento. Ele chega a comer até 30 mil insetos por dia!

Comumente, o tamanduá-bandeira é um animal pacífico. Mas, se for preciso se defender, ele pode usar seu poderoso abraço. Diante do perigo, ele se apoia em suas patas traseiras, mantém os braços abertos e mostra as garras para o inimigo, esperando pelo ataque. Quando é agredido, ele abraça e crava suas unhas nas costas do predador de uma maneira tão forte, que pode até matá-lo. Daí vem a expressão “abraço de tamanduá”.

**Texto expositivo: Os Tamanduás**

1. *Onde vive o tamanduá?*

Resposta:__________________________________________________________________________________________________________________________________

*2. Do que se alimenta o filhote do tamanduá-bandeira?*

Resposta:__________________________________________________________________________________________________________________________________

*3. Em que época do ano você acha que os tamanduás se acasalam?*

Resposta:_____________________________________________________________*_____________________________________________________________________*

*4.Sabendo que há quatro estações no ano, você acha que o tamanduá-bandeira é um animal solitário? Justifique.*

Resposta:__________________________________________________________________________________________________________________________________

*5. Como é o focinho do tamanduá-bandeira?*

Resposta:__________________________________________________________________________________________________________________________________

*6. Como é a língua do tamanduá-bandeira?*

*Resposta:__________________________________________________________________________________________________________________________________*

*7. Que ferramentas o tamanduá-bandeira tem e usa para se alimentar?*

Resposta:__________________________________________________________________________________________________________________________________

*8. Dê o nome de dois bichos que o tamanduá-bandeira come.*

Resposta:_____________________________________________________________*_____________________________________________________________________*

*9. Por que você acha que o tamanduá-bandeira é um animal pacífico?*

Resposta:__________________________________________________________________________________________________________________________________

*10. Baseado no texto, quantos filhotes você acha que a fêmea tamanduá-bandeira tem por ano?*

*Resposta:__________________________________________________________________________________________________________________________________*

*11. Qual o significado da expressão ‘abraço de tamanduá’?*

Resposta:__________________________________________________________________________________________________________________________________

*12. Você consideraria o tamanduá-bandeira um animal cruel? Justifique.*

Resposta:__________________________________________________________________________________________________________________________________

*13. Em que situação o tamanduá-bandeira fica agressivo?*

Resposta:__________________________________________________________________________________________________________________________________

*14. Em que situação você diria que recebeu um abraço de tamanduá?*

*Resposta:__________________________________________________________________________________________________________________________________*

*15. Um forte abraço de mãe pode ser considerado um abraço de tamanduá? Por que?*

Resposta:__________________________________________________________________________________________________________________________________

|  | **Tipo de Questão** | **Análise da resposta** | | | |
| --- | --- | --- | --- | --- | --- |
| **1.** | Literal | ( ) acerto ( ) erro | | | |
| **2.** | Gap – Filling | ( ) acerto ( ) erro | | | |
| **3.** | Gap – Filling | ( ) acerto ( ) erro | | | |
| **4.** | Text – Connecting | ( ) acerto ( ) erro | | | |
| **5.** | Literal | ( ) acerto ( ) erro | | | |
| **6.** | Literal | ( ) acerto ( ) erro | | | |
| **7.** | Text – Connecting | ( ) acerto ( ) erro | | | |
| **8.** | Text – Connecting | ( ) acerto ( ) erro | | | |
| **9.** | Text – Connecting | ( ) acerto ( ) erro | | | |
| **10.** | Gap – Filling | ( ) acerto ( ) erro | | | |
| **11.** | Gap – Filling | ( ) acerto ( ) erro | | | |
| **12.** | Modelo de Situação | ( ) acerto ( ) erro | | | |
| **13.** | Text – Connecting | ( ) acerto ( ) erro | | | |
| **14.** | Modelo de Situação | ( ) acerto ( ) erro | | | |
| **15.** | Modelo de Situação | ( ) acerto ( ) erro | | | |
| **TOTAL DE ACERTOS** | | **___ LIT** | **___ TC** | **___ GF** | **___ MS** |

**Pontuação:**

| **Pontuação Total: _________** |
| --- |

Literal = 1 ponto

Text-Connecting = 2 pontos

Gap-Filling = 3 pontos

Modelo de Situação = 4 pontos
